# Significant association of MTHFD1 1958G>A single nucleotide polymorphism with nonsyndromic cleft lip and palate in Indian population

**DOI:** 10.4317/medoral.19796

**Published:** 2014-08-17

**Authors:** Jyotsna Murthy, Venkatesh B. Gurramkonda, Bhaskar VKS. Lakkakula

**Affiliations:** 1 Department of Plastic Surgery, Sri Ramachandra University, Chennai, India; 2 Department of Biomedical Sciences, Sri Ramachandra University, Chennai, India; 3 Sickle Cell Institute Chhattisgarh, Raipur, India

## Abstract

Objectives: Nonsyndromic cleft lip and palate (NSCLP) is genetically distinct from those with syndromic clefts, and accounts for ~70% of cases with Oral clefts. Folate, or vitamin B9, is an essential nutrient in our diet. Allelic variants in genes involved in the folate pathway might be expected to have an impact on risk of oral clefts. Given the key role of methylenetetrahydrofolate dehydrogenase 1 (MTHFD1) in folate metabolism, it would be of significant interest to assess its role in NSCLP etiology. Study Design: The present study aims at examining the association between MTHFD1 1958G>A polymorphism and NSCLP risk by conducting a case-control study in south Indian population. Our sample comprised of 142 cases with nonsyndromic clefts and 141 controls without clefts or family history of clefting. The MTHFD1 1958G>A polymorphism was genotyped using PCR-RFLP. Results: An increased risk was found for the heterozygous 1958GA (OR=2.44; P=0.020) and homozygous 1958AA (OR=2.45; P=0.012) genotypes in the children. When the dominant model (AG+AA vs GG) was applied the risk remained the same as co-dominant model, but the level of significance increased (OR=2.44; P=0.002). Conclusions: The results indicated the MTHFD1 1958G>A polymorphism to be one of the important genetic determinants of NSCLP risk in South Indian subjects.

** Key words:**MTHFD1, orofacial cleft, SNP, genetics.

## Introduction

Nonsyndromic cleft lip and palate (NSCLP) is genetically distinct from those with syndromic clefts, and accounts for ~70% of cases with Oral clefts. The etiology of NSCLP is multifactorial, with both genetic and environmental factors, involving complex gene-gene and gene-environment interactions, and it is these interactions that play a critical role (1). Folate, or vitamin B9, is an essential nutrient in our diet. Folate metabolism provides one-carbon building blocks for the synthesis of nucleic acid bases. Folate coenzyme is essential for the synthesis of methionine and methionine is required for the synthesis of the universal methyl donor S-adenosylmethionine (2). A significant number of hypotheses have been published regarding the critical role played by the folate during preconception, conception, 

implantation, placentation and embryo or organogenesis stages in the manifestation of birth defects. Evidence from epidemiologic studies have conclusively shown that the prenatal folic acid supplementation reduces risk of many congenital anomalies (3).

MTHFD1 is one of the important genes that is involved in folate metabolism. MTHFD1 gene encodes trifunctional enzyme 5,10-methylenetetrahydrofolate dehydrogenase; 5,10-methenyltetrahydrofolate cyclohydrolase and 10-formylotetrahydrofolate synthetase (4). This enzyme catalyzes the conversion of 1-carbon derivatives of tetrahydrofolate (THF) to form the cofactor 10-formylTHF, which serves as a one-carbon donor for the de novo biosynthesis of purines (5). 5,10-methyleneTHF that is pro-duced from the condensation of serine and THF is utilized in the de novo synthesis of thymidylate or otherwise, can be irreversibly reduced by MTHFR to 5-methylTHF, which is involved in the methylation of homocysteine (6,7).

The MTHFD1, gene is located on chromosome 14q23.3 and spans 71 kb length with a total of 28 exons. Previous studies have reported the association of MTHFD1 gene variants with serum folic acid and homocysteine levels (8,9). The common G1958A SNP, which is located in exon 20 of MTHFD1 gene is associated with folate-mediated pathologies such as congenital anomalies (neural tube defects, heart defects, oral clefts) and several cancers (10). As the MTHFD1 is a potential candidate gene for investigation in relation to cleft palate risk, and since the previous studies have provided contradictory results (11-14), the present case-control study was undertaken to examine the association between MTHFD1 1958G>A and nonsyndromic cleft lip and palate in south Indian population.

## Material and Methods

- Subjects

The sample consisted of 283 individuals ascertained from Cleft and Craniofacial Centre, Sri Ramachandra University, Chennai, India. All cases were evaluated by two different plastic surgeons for their individual phenotypic features, and was also cross verified through their medical records. There is no involvement of oral pathologist in this study. The case group comprised of 142 individuals with NSCLP (123 CLP: cleft lip with or without cleft palate + 19 CPO: cleft palate only). The control group was recruited from the same region, included 141 unrelated individuals without clefts or family history of clefting in three generations. The subjects with congenital malformations or major developmental disorders were excluded from the study. The study was approved by the Institutional Ethics Committee of the Sri Ramachandra University, Chennai, India, and all the study subjects gave informed consent. As many of the children were minors, the consent was obtained from their parents or legal guardian.

- Genotyping

From each study subject 3 ml blood sample was collected into an EDTA vacutainer. Genomic DNA was isolated from leukocytes using phenol-chloroform extraction and ethanol precipitation (15). MTHFD1 1958G>A (rs2236225) SNP genotyping was performed following polymerase chain reaction-restriction fragment length polymorphism method (16). Briefly, 310 base pairs (bp) fragment of MTHFD1 1958G>A region was amplified with the primers of 5’-CCT GGT TTC CAC AGG GCA CTC-3’ and 5’-CCA CGT GGG GGC AGA GGC CGG AAT ACC GG -3’. The PCR amplicons were incubated with the MspI restriction enzyme at 37oC for 4 hours and the digested products were resolved by electrophoresis on 3% agarose gel. Upon digestion, 310-bp PCR product cleaved into two fragments of 282-bp and 28-bp for the A allele and in the case of G allele the 310-bp PCR product cleaved into three smaller fragments of 196-bp, 86-bp and 40-bp. The digested products were visualized under UV light by two researchers and independently scored the genotypes to minimize errors.

- Statistical Analysis

Allele frequencies were calculated by direct counting of alleles. Hardy-Weinberg equilibrium (HWE) analysis was performed by comparison of observed and expected genotype frequencies using chi-squared goodness-of-fit test. The association between different cleft phenotypes and MTHFD1 1958G>A polymorphism was analyzed by χ2-test. To assess the possible association with NSCLP, the comparative analysis for the MTHFD1 1958G>A polymorphism distribution was performed for the whole group of NSCLP (n=142), as well as for the subgroups of patients with CLP (n=123), and CPO (n=19). The same control group (n=141) was considered for all the three case groups. The risk estimates were calculated with the wild type as the reference category. The effect of the 1958G>A variant was examined in 3 hypothetical risk models, co-dominant, dominant (GA+AA vs.GG) and recessive (GA+GG vs.AA) models. The statistical analysis was performed using SPSS statistical software version 16.0 (SPSS Inc, Chicago, Illinois) for Windows. A two-sided p-value <0.05 was considered to be statistically significant. From the HapMap populations 20kb up and downstream SNPs around rs2236225 were extracted and LD maps were constructed using Haploview (17).

## Results

**Figure 1:**
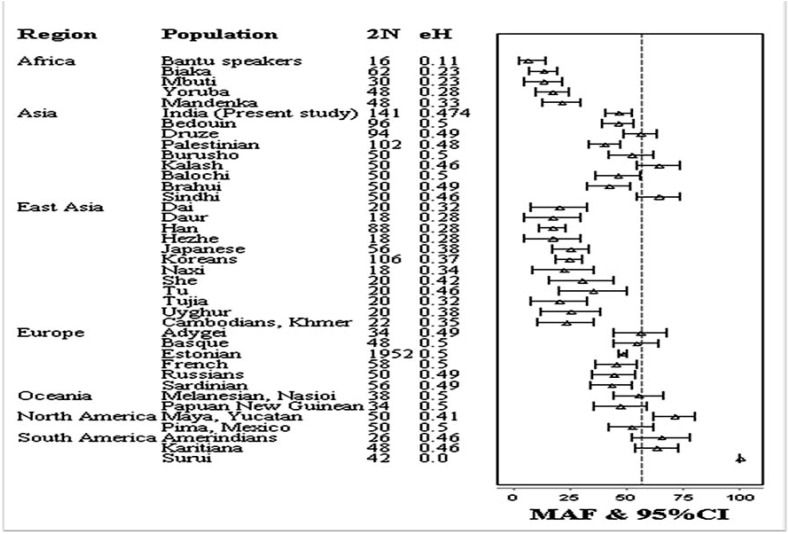
The frequency of MTHFD1 1958G>A SNP in the current study compared to the world populations. Forest plot represents minor allele frequency (MAF) with 95% confidence interval (CI). The frequency data are the same as in ALFRED (http://alfred.med.yale.edu/alfred/SiteTable1A_working.asp?siteuid=SI315597E). 2N: sample size; eH: expected heterozygosity.

The distribution of the MTHFD1 1958G>A variant genotypes and alleles in both cases and NSCLP groups are presented in  Table 1. The proportions of genotypes were 14.8 % GG, 58.5 % AG, 26.8 % AA in cases and 29.8 % GG, 48.2 % AC, 22.0 % CC in controls. The A allele frequency was 56.0% in cases and 46.1% in controls. The genotype distribution in the control group followed HardyWeinberg equilibrium (*P*=0.726). Significant difference in allele frequencies were found between control and the NSCLP groups ( Table 2). OR and 95% CI were calculated to assess the impact of the MTHFD1 1958G>A variant on NSCLP risk ( Table 2). Significantly increased NSCLP risk was found for both heterozygous (OR=2.44; 95% CI: 1.32-4.51;* p*=0.004) and homozygous (OR=2.45; 95% CI: 1.20-4.97; *p*=0.012) genotypes. Increased NSCLP risk was also found under dominant (AG+AA vs GG; OR=2.44; 95% CI: 1.36-4.30; *p*=0.002) and allelic models (A vs. G; OR = 1.49; 95% CI: 1.07-2.07; *p*= 0.018). The recessive effect of the 1958A variant (AA vs GG+AG) in cases was not significant (OR=1.30; 95% CI: 0.75-2.24; *P*=0.350). In subgroup analysis, the 1958A variant showed a similar trend of association for CLP group ( Table 2). The MTHFD1 1958G>A variant did not show any significant association with CPO risk under three different models ( Table 2). The MTHFD1 1958G>A variant allele frequencies representing different world populations were presented in figure 1. The MTHFD1 1958G>A minor allele frequency and expected heterozygosity in Indian populations showed little proximity with the other Asian, European and American populations, but it was quite different from East Asian and African populations (Fig. 1).

**Table 1:**
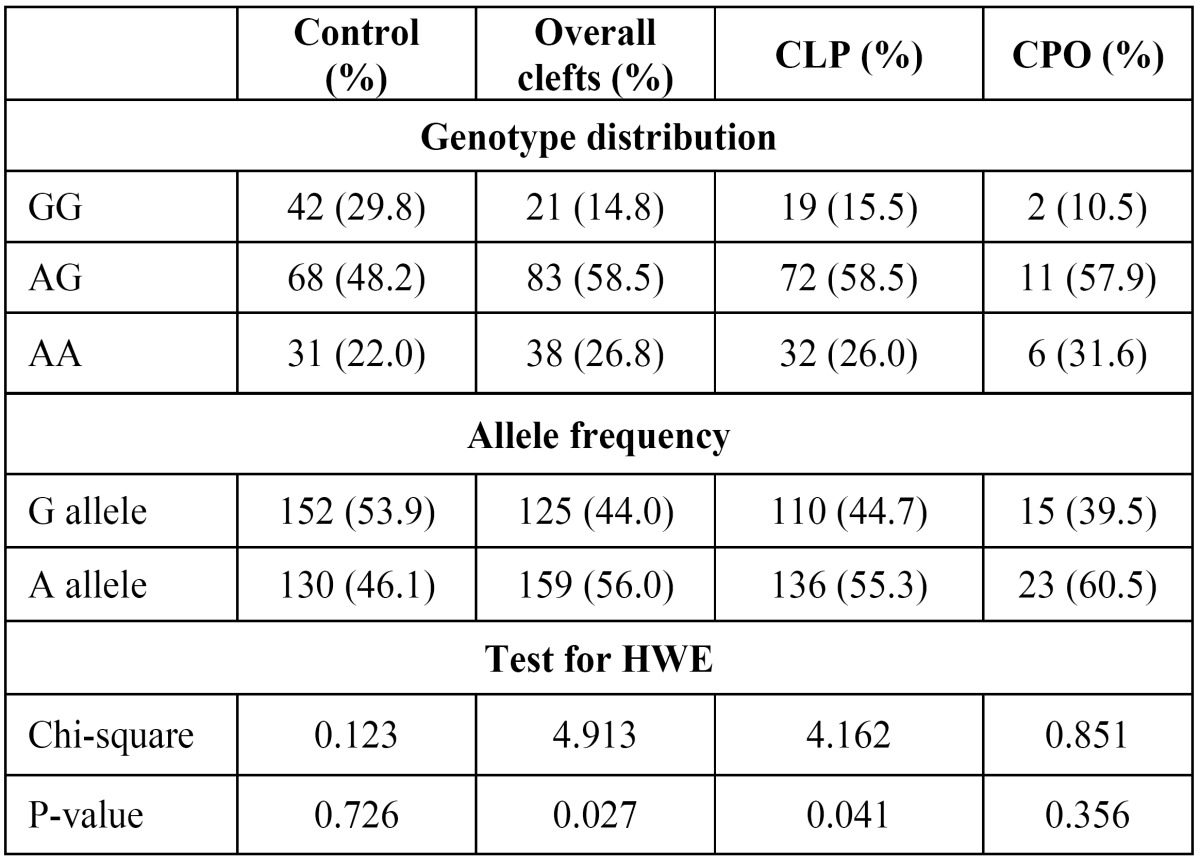
Genotype distribution and allele frequencies of the MTHFD1 1958G>A SNP in cleft lip and palate.

**Table 2:**
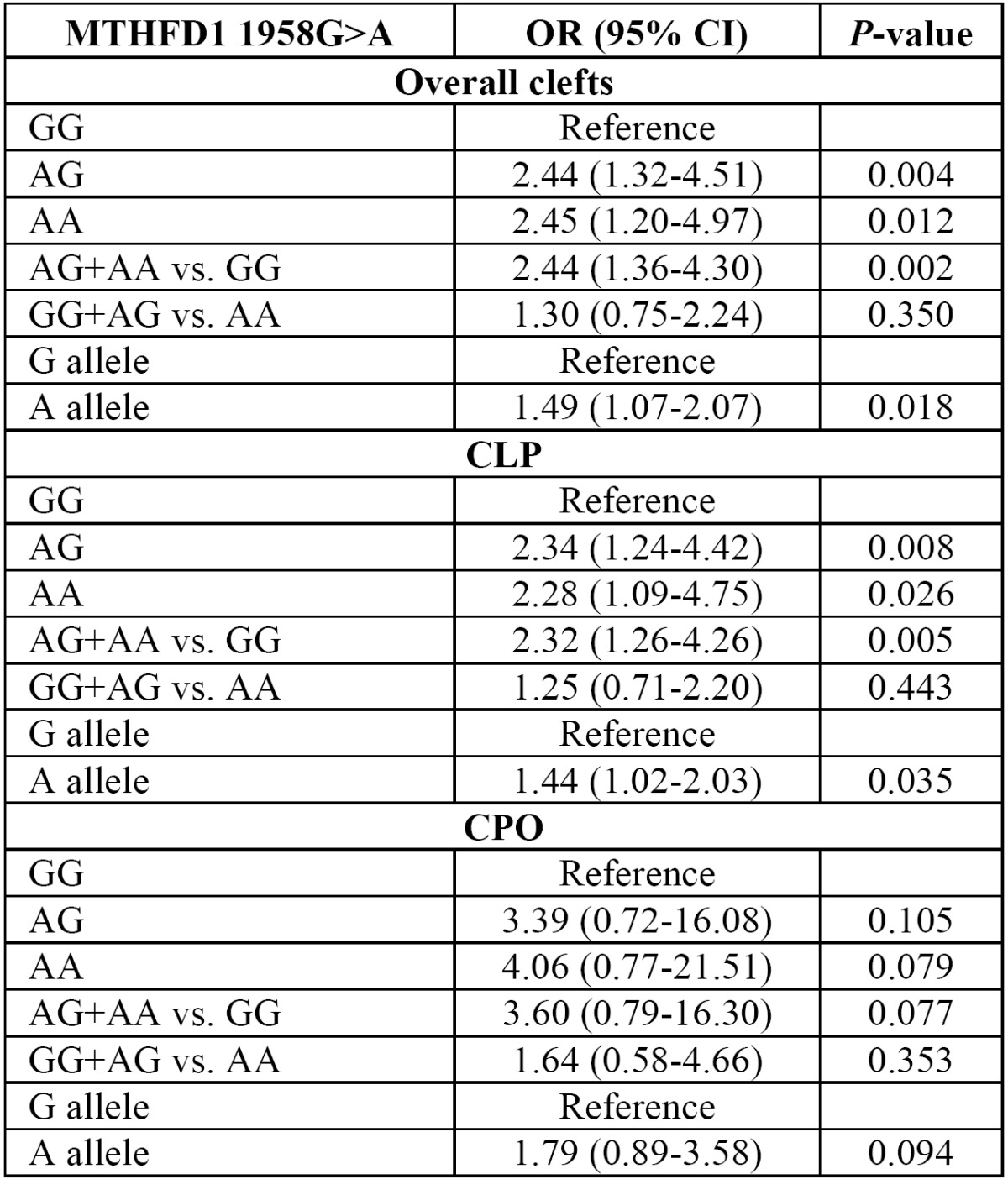
Results of association tests with MTHFD1 1958G>A SNP in cleft lip and palate.

## Discussion

The MTHFD1 1958G>A minor allele frequency (A allele) in our controls was 46.1%, which is analogous to the frequency of 47% found in the GIH population of HapMap (http://hapmap.ncbi.nlm.nih.gov). The frequency reported in the current study is slightly lesser than that (55%) found in a small sample of south Indians (18). This discrepancy may be due to the variations in source of samples included in these studies. The MTHFD1 1958G>A polymorphism has significantly increased the risk of developing cleft lip and palate in cases that inherit one copy or two copies of this polymorphism. The magnitude of risk (OR) is almost similar when both genotypes were analyzed separately (GA vs. GG=2.44; AA vs. GG=2.45) and also in a dominant genetic model of action (AG+AA vs. GG=2.44).

As MTHFD1 is involved in folate pools and folate-dependent reactions, its gene variants have been studied extensively for their association with birth defects in humans (9,19,20). The results evidenced the association between the MTHFD1 1958G>A polymorphism and NSCLP to be convincingly powered and it is not in agreement with the earlier studies in Polish (11,12), Norwegian (13) and Italian populations (14). Analysis of MTHFD1 1958G>A variant in mothers with CLP children as well as control mothers revealed the absence of statistical differences in allele and genotype frequencies (12,13). MTHFD1 1958G>A has not contributed to NSCLP risk in the Polish population separately or by showing epistatic interaction with other variants in genes of choline and folate metabolism (11). Analysis of MTHFD1 A1958G and G401A polymorphisms in 216 CLP triads of Italian origin revealed relatively low linkage disequilibrium between these markers and the alleles or haplotypes are not associated with CLP risk (14). In a sample of Irish population MTHFD1 1958G>A variant did not show any association between the A allele and CPO case status, but CPO case mothers were significantly more likely to be AA compared to controls. Further, in both case-control and mother-control analyses the variant allele is associated with increased risk of CLP (21). Gene-gene interaction analysis showed that the MTHFD1 1958G>A variant in combination with MTHFR rs2274976 and SLC19A1 rs1051266 predicted the maternal risk for NSCLP in the Brazilian population (22). Both TRIMM and HAPLIN methods that were used to detect multi-marker effects on oral clefts of Norway and Denmark failed to detect the maternal effects of MTHFD1 gene variants (23).

**Figure 2:**
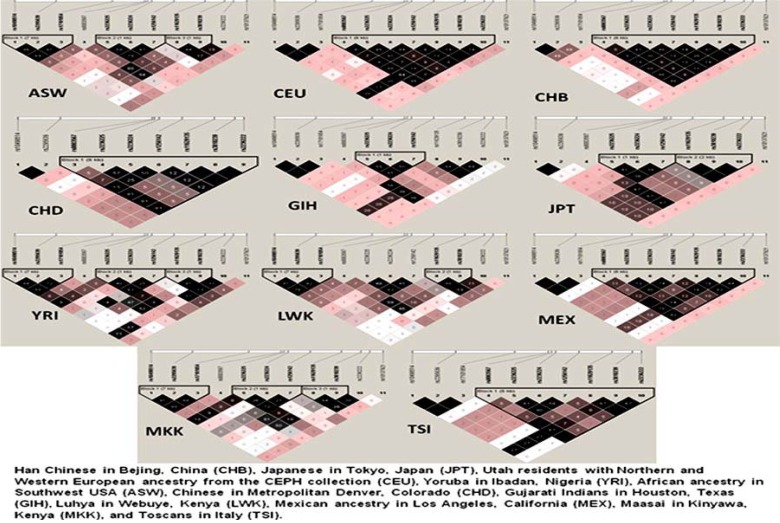
Linkage disequilibrium profiles in different world populations studied in International HapMap Project. Colour coding represents the D'/LOD values and the values in cells are r2 multiplied by 100.

There is no consistent evidence to show that the MTHFD1 1958G>A polymorphism affected MTHFD1 function or homocysteine levels. The 1958G>A SNP of MTHFD1 results in the substitution of a glutamine (Gln) for arginine (Arg) within the 10-formylTHF synthetase domain of the MTHFD1 enzyme (5). When the orthologs of MTHFD1 in some species is considered, this residue is conserved and contains arginine (rat and mouse) or lysine (prokaryotes, insects, plants), substitution of this amino acid with a glutamine may interfere with the structure and function and thereby causing disturbances in the folate status or homocysteine levels (9,24). Under normal assay conditions, this polymorphism had no effect on synthetase activity but it was shown to reduce enzyme stability and inhibition of de novo purine biosynthesis (25). On the other hand, this variant may be in LD with a different, as yet unstudied, variant that modulates MTHFD1 function. Analysis of 20kb up and downstream SNPs around rs2236225 from the HapMap data demonstrated that the European (CEU, MEX and TSI) and Asian (JPT, CHB, CHD and GIH) populations formed the single large LD block. The African populations exhibited weak LD and formed two to three small LD blocks. Except in east Asian populations, the rs2236225 is found to be in strong LD with other intronic SNP rs1256142 (Fig.2).

In summary, the current study demonstrates a strong association between MTHFD1 1958G>A and risk of NSCLP in south Indian population. Further the relationship between additional mutations in the MTHFD1 gene and serum folate levels or plasma homocysteine levels and their interactions could be examined to provide an eligible conclusion.
